# Understanding and Breaking the Intergenerational Cycle of Abuse in Families Enrolled in Routine Mental Health and Welfare Services by Investigating the Feasibility and Effectiveness of a Mentalization-Based Early Intervention Program (UBICA-II Study): Study Protocol for a Non-Randomized, Open-Label, Single-Arm Feasibility Study

**DOI:** 10.3390/children11030267

**Published:** 2024-02-20

**Authors:** Denise Dittmann, Astrid Dempfle, Anke Nießen, Ira Puchert, Kerstin Konrad, Beate Herpertz-Dahlmann

**Affiliations:** 1 Department of Child and Adolescent Psychiatry Psychosomatics and Psychotherapy of the RWTH Aachen, Neuenhoferweg 21, 52074 Aachen, Germany; 2 Department of Child and Adolescent Psychiatry Psychosomatics and Psychotherapy of the RWTH Aachen, Child Neuropsychology Section, University Hospital Aachen, Neuenhoferweg 21, 52074 Aachen, Germany; 3 Institute of Medical Informatics and Statistics, Kiel University and University Hospital Schleswig-Holstein, 24118 Kiel, Germany; 4 JARA-Brain Institute II, Molecular Neuroscience and Neuroimaging, RWTH Aachen & Research Centre, 52428 Juelich, Germany

**Keywords:** transgenerational transmission of abuse, mentalization-based integrative team approach, early prevention program plus

## Abstract

Although home visiting programs have generally shown small overall effects on the prevention of child maltreatment, at-risk families with severe strain do not seem to benefit sufficiently from this support. A crucial factor for success seems to be the quality of the service system. The aim of the current study is to evaluate the effects of mentalization-based team supervision on the already existing welfare service of a German early prevention program (EPP). This will be a non-randomized, open-label, single-arm feasibility study. The EPP staff will be trained according to the mentalization-based team approach (MB-TA) and regularly receive MFT supervision by a trained and experienced child and adolescent psychiatrist. A minimum of eighty-four families with defined risk factors with children below 24 months of age and pregnant women in the third trimester will be included. Assessments will take place at T0 (after inclusion in the study), at T1 (after family care ends, as an intermediate assessment,) and at T2 (as a follow-up). We hypothesize that the risk of maltreatment can be reduced by strengthening the skills and capacities of the primary care system. This will be evaluated at the end of the follow-up period by comparing the Parental Stress Index (PSI) scores of all participants pre- and postintervention. Stress levels and mentalization abilities will be assessed as feasibility endpoints for the participating EPP teams.

## 1. Introduction

Many different risk factors for the occurrence of maltreatment in burdened families have been identified. Most of these risk factors have been found at the parental level [1]. For instance, low parental education and low family income levels have been shown to be risk factors for child abuse and neglect [2]. Additionally, parents with their own experiences of early life maltreatment (ELM) are more likely to maltreat their children, potentially leading to an intergenerational cycle of abuse and neglect [3,4,5]. Thereby, various pathways may explain the transmission, including learning theory, disorganized attachment, epigenetic aspects, and intergenerational transmission of psychopathology [6]. A recent cross-sectional study underpinned these previous findings by examining the transmission across three generations, grandparents, parents, and young adults, and found continuity for maltreatment across all three generations [7]. A meta-analysis of 84 studies provided a qualitative summary of the current knowledge regarding this cycle. These results indicated that the risk of child maltreatment in families in which the parents experienced ELM was almost three times higher than that in families in which the parents did not experience ELM [6]. There is also evidence that socially isolated parents may be more prone to maltreating their children [8]. Parenting stress, in general, is another identified risk factor for child abuse and neglect. Crum and colleagues conducted a study to assess the relationships between parental stress, abuse potential, and social and behavioral outcomes in children. The results demonstrated that parental stress was consistently linked to abuse potential, supporting the association already determined in previous research [9]. Parents with mental illness are also at an increased risk of maltreating their children [10]. In a recent study, persistent maternal depressive symptoms mediated the relationship between maternal history of maltreatment and current parenting stress at the 18-month assessment during a home-visiting prevention program [11]. Mothers with depression were much more likely to have a child for whom maltreatment was reported than mothers without depression [12]. Other psychiatric disorders, such as borderline personality disorder [13] or substance abuse, are also associated with an increased risk of parental ELM and child abuse. Overall, parents with a high psychosocial and economic burden are at risk of maltreating their children and prematurely dropping out of support programs that aim to prevent child abuse and neglect [14]. Early dropout represents a particular challenge for such programs. Special attention must be paid to these hard-to-reach clients to ensure effective support.

In Germany, early prevention programs aim to improve the parent–child relationship and thereby try to reduce the risk of maltreatment. Some studies have examined the effectiveness of such early prevention programs. Taubner [15] carried out a meta-analysis on the effectiveness of early providing preventive help in Germany. Child development outcomes (mental and physical) and the effects on maternal characteristics (symptom burden, parenting skills, and social support) were considered the primary outcome criteria. The results showed that the implemented programs had small positive effects on the symptom burden of the participating mothers but no effects on maternal competency development. At the child level, the data did not reveal a measurable benefit in terms of psychological (social and emotional) development for the participating children. A longitudinal pilot study from 2016 also showed that the EP clientele represents a high-risk group but that the intervention did not contribute to an improvement in the child’s developmental status. More precisely, the results revealed that the child’s cognitive development was predicted by both maternal sensitivity and the mother’s psychosocial stress, but the amount, type, and intensity of the EPP did not have an effect on the child’s development [16]. Overall, there are many different program approaches in Germany that were rarely evaluated in terms of their effectiveness [17]. However, the programs that were evaluated showed only small effects and varied considerably between different program approaches [18]. This illustrates the urgent need for further research in the field of early intervention programs and their continuous improvement.

International research also reports strongly fluctuating and rather low effects of EPPs. However, more and more studies have indicated which factors are most likely to be associated with program success. In a systematic review of RCTs with interventions designed to reduce child abuse in high-risk families, in contrast to other outpatient services, only home visitations had significant evidence to reduce child abuse [19]. Further studies prove that programs with more frequent home visits [20] and targeted prevention approaches appear to be superior to universal approaches [21]. However, the findings varied considerably. In a meta-analysis of studies by Chen and Chan assessing the effects of parenting programs (including home visits) on child maltreatment prevention [8], 37 studies met all the quality criteria defined by the authors. These programs significantly reduced risk factors and enhanced protective factors (including positive parenting attitudes, parent–child interactions, positive parenting behaviors, and parental confidence and satisfaction) associated with child maltreatment. Another large meta-analysis of 33 home-visiting programs for the prevention of child maltreatment demonstrated that the most cost-effective programs used “professional home visitors in a multidisciplinary team, target high-risk populations and include more than just home visits”, e.g., have contact with other welfare agencies and the health care system [22]. A meta-analysis of 156 studies with nine different home visiting programs aimed at reducing child maltreatment, supervision, and training emerged as important implementation factors [23]. Further studies confirmed the importance of supervision [24] and that multiprofessional teams with appropriate training quality are superior to paraprofessional teams [25]. Despite emerging evidence about the health benefits and reduced risk of maltreatment and neglect achieved through home visiting programs [13,26], a crucial factor for success is the quality of the service, including multi-professional teams, cooperation with community health and welfare services, and regular supervision.

Bateman and Fonagy [27] developed a therapeutic strategy called mentalization-based treatment (MBT) (“mentalizing is the process by which we make sense of each other and ourselves in terms of subjective states and mental processes”), e.g., “we form beliefs about the mental states of those with whom we interact and our own mental states are strongly influenced by these beliefs.” Mentalization is seen as a key factor in personality and social functioning, promoting resilience and mental health. More recently, the MBT approach was used for health care professionals, aiming to provide general practitioners with MBT skills for use in everyday settings [28]. These professionals perceived MBT skills training as promoting empathy and understanding for their clients and increasing consistency in their teamwork. Moreover, they felt empowered and more confident in working with hard-to-reach patients or clients [28]. In the next step, this approach was expanded to teams that worked with patients suffering from severe and complex mental health problems, such as adolescents with self-harm, borderline symptoms (Adolescent Mentalization-Based Integrative Treatment, AMBIT), and substance abuse [29,30]. The AMBIT approach, first published by Bevington et al. [31] and since refined in [29,30], comprises four core techniques: “working with your client”, “working with your team”, “working with your network”, and “learning as a team.” It suggests that professionals working with mentally ill, burdened, and hard-to-reach clients should attend to their team and network relationships at least as much as they attend to their relationship with the client [27]. This approach applies the principle of mentalization to relationships with clients, other team members, and cooperating agencies and helps staff develop empathy, flexibility, and a more consistent approach to difficult patients. In Germany, the EPP supports at-risk families in coping with everyday life and childrearing issues. Although the EPP involves a well-connected, multi-professional team that provides home visits, families with extreme stresses and strains do not seem to benefit sufficiently from this support [32]. In the present project, we will implement an approach similar to the AMBIT approach (see above), which is mainly characterized by MBT supervision carried out by experienced child and adolescent psychiatrists. In the three years preceding the study, approximately sixty-nine percent of the EPP clients stayed in treatment for less than three months, indicating major problems for the continuity of support. Built on participatory approaches, meetings with the EPP teams in Aachen and surrounding areas were conducted prior to the planning of the trial to optimally address professionals’ needs. They complained about a lack of knowledge in psychiatry/child psychiatry, which made it difficult for them to recognize mental disorders in parents, although more than a quarter of their clients contacted them due to severe parental mental health problems [33]. We aim to perform the following: (1) help EPP professionals mentalize with hard-to-reach clients and thereby better motivate these parents to stay in the care of the EPP, which is hypothesized to result in a better understanding of parental mental states and a more effective home visiting program; (2) evaluate the association between parental risk factors (including ELM) and the effectiveness of care by the “EPP-plus” (e.g., including MBT); and (3) investigate the feasibility of MBT implementation by exploring the acceptance and workload of the EPP team members, We hypothesize that the intervention will help professionals to better consider parents’ perspectives and to communicate more effectively with their team members and other institutions for child protection.

## 2. Methods

### 2.1. Objectives

The main objective of this trial is to investigate the feasibility of adding a mentalization-based integrative team approach (MB-TA) to the EPP to better capture the risk of maltreatment among hard-to-reach clients in the existing EPP. EPP staff will be trained according to the MBT approach and regularly receive mentalization-based team supervision by a trained and experienced child and adolescent psychiatrist. The aim is to support staff in developing a more empathetic and consistent approach to challenging clients to keep them in the care program and thus reduce the risk of child abuse, maltreatment, and neglect. We hypothesize that the risk of maltreatment can be reduced by strengthening the competencies of professionals in the primary care system. This will be evaluated at the end of the follow-up period by comparing the subjects’ burden associated with the care for their child (measured by the Parental Stress Index scores) pre- and postintervention.

Secondary objectives:

The secondary objective is to investigate the modulating role of parental history of ELM in treatment success. This will be evaluated by analyzing the association between ELM (measured by the childhood experience of care and abuse questionnaire, CECA.Q) and the values on the primary outcome (Parental Stress Index).

Moreover, the feasibility of the approach will be investigated based on the acceptance of the intervention by staff members and their subjective feelings about the associated additional workload. This will be evaluated by measuring staff stress levels (using the trier inventory for chronic stress and a burden questionnaire) and staff metallization abilities (using the AMBIT service evaluation questionnaire).

### 2.2. Study Design

The current trial is a single-center, non-randomized, open-label, single-arm feasibility study, including two local EPP teams in the wider area of Aachen, Germany. It is part of a larger consortium (the UBICA-II study), including an MBT-based intervention trial for psychiatric patients who are parents [34]. A total of at least 84 families in the care of the EPP will be systematically assessed at three time points: at the beginning of support from the welfare system (preintervention; T0), when support ends (postintervention; T1), and when the child is 12, 18, 24, or 30 months old, depending on their age at the time of inclusion in the study (follow-up; T2). The trial was approved by the local Research Ethics Committee at the University Hospital RWTH Aachen (reference EK 221/19) and will be carried out in accordance with the Declaration of Helsinki. The study was registered at the German Clinical Trials Register (DRKS00022075 on 8 July 2020). The study protocol was adapted in 2020, when it turned out that, due to the COVID-19 pandemic, the originally planned non-randomized study design with the nonconcurrent recruitment of two groups (first, a control group receiving usual EPP support, followed by the training and subsequent recruitment of the EPP employees for the “EPP-plus” intervention (experimental condition)) would not lead to comparable group conditions. Thus, the study was modified to a non-randomized, single-arm feasibility study. The aims of the feasibility study were adapted and now include evaluating (1) the acceptance of the approach by the EPP staff, (2) the burden of the EPP staff, and (3) the success of the EPP staff to better retain hard-to-reach parents/caregivers in the care program, in addition to our measures for the risk of maltreatment by parents. We chose a non-randomized, single-group feasibility study design for the following reasons: the EPP support systems vary across Germany with respect to type, dose, and mode of interventions, professional backgrounds, frequency of supervision and networking of the team, and legal and financial situations. Thus, it is not possible to choose a (non-randomized) comparable concurrent control group from another geographical region. Due to the planned team intervention, the randomization of individual staff members or individual parent dyads would have been impossible. The possible alternative approach of a cluster-randomized controlled trial with randomized teams would have resulted in very large sample sizes, e.g., for an effect size of d = 0.4, we would need 26 clusters with approximately 30 families each (total of 780 families) or approximately 23 clusters with 50 families each (total 1180 families), with an expected intracluster correlation coefficient of 0.1, entailing huge costs and logistical efforts. Since neither the feasibility nor estimated effect sizes on parents have been investigated for this mentalization-based team approach before, our current study is a necessary first step before such a large cluster-randomized trial.

### 2.3. Study Setting

The trial is being performed at the Department of Child and Adolescent Psychiatry, Psychotherapy and Psychosomatics, University Hospital RWTH Aachen. Data collection takes place via home visits. The EPP teams from Aachen and Frechen, a small town near Aachen, are participating in the study. Both sites are located in Germany. The study is part of the UBICA-II consortium with researchers from Berlin and Heidelberg (see https://www.ubica.site/tp_fruehehilfen.html (accessed on 2 January 2024) for more information). The Coordination Centre for Clinical Trials (KKS) in Heidelberg monitors the data, and the Institute of Medical Informatics and Statistics (IMIS) in Kiel is responsible for statistical planning and analysis.

### 2.4. Eligibility Criteria

We identified two EPP teams in Aachen and the surrounding areas with comparable team structures and professional backgrounds (i.e., family midwives, social workers, or pediatric nurses). These teams are typical of EPP teams in either rural or urban German regions. The majority of staff members in the teams have to provide informed consent to participate in the trial. We consecutively include all families (mothers or fathers and their children) seeking support in the EPP. The inclusion and exclusion criteria are summarized in Table 1. The risk factors were previously identified according to a large sample of 837 at-risk families [33]. Parents of children aged ≤ 24 months or women in the third trimester of pregnancy will be included at the start of the intervention. Participants must provide informed consent for data collection within the trial. No other exclusion criteria will be used to achieve maximum generalizability and representativeness. In families with insufficient knowledge of German, data collection will be carried out with the help of a translator. Support by the EPP is available for families who are not participating in the study. However, the intervention of the EPP corresponds to the “EPP-plus” approach for nonparticipating families as well since the entire team has been trained in the MB-TA. Thus, inclusion in the study only means that additional data will be collected from parents, not that a different intervention will be provided.

### 2.5. Intervention Description

#### 2.5.1. Intervention: MB-TA

With the implementation of the “EPP-plus” intervention, the standard care offered by the EPP will be augmented by the following elements: (1) training of staff to recognize mental disorders and the risk of maltreatment of parents and children and (2) an MB-TA approach developed for work with hard-to-reach clients, which applies to relationships with clients, team members and work across agencies. The training will consist of a two-day training session offered by Prof. Dr. Taubner and her team. A one-day refresher training session is offered every 1.5 years. The training will be supported by regular case conferences and biweekly staff supervision by experienced child and adult psychiatrists/psychotherapists. The elements in the supervision will be raising awareness for reasons of problems in relationship building, learning techniques of appreciative, open conversation, recognizing one’s own mentalization difficulties, applying techniques to stabilize and restore one’s own mentalizing, using techniques to promote the mentalization of parents, recognizing relationship breaks, and knowing techniques to actively deal with relationship breaks and repairing them. All supervisors will have been trained at the Anna Freud Center in London and attended the “Mentalization-Based Treatment for Families” course. The supervisors, in turn, will receive ongoing supervision (meta-supervision), again provided by Prof. Taubner and her team. Standardized psychiatric diagnostics will be systematically assessed at all three time points by trained psychologists. At T0, standardized interviews on psychiatric disorders will be conducted with the parents. If the caregivers give separate consent, these diagnostic results will be made available to the respective supervisor and can be included in the next supervision session. As outcome measure preintervention scores will be compared with postintervention scores to measure the effects of the intervention on the subjects. In the preintervention period, the EPP professional may have visited families ideally a maximum of 5 times (corresponding to T0). The end of the intervention will be defined by the end of support by the EPP (corresponding to T1).

#### 2.5.2. Criteria for Discontinuing or Modifying Allocated Interventions

The implemented team approach applies to every team member and cannot be changed or modified individually. However, if necessary, the organizational framework of the intervention can be adjusted; for example, if the EPP teams complain that the regular supervision sessions are too time-consuming, they should be done at longer intervals, or vice versa. Side effects of the intervention will be assessed via the occurrence of adverse events. If single adverse events occur, these will be discussed individually with the respective EPP employee. If there are several adverse events that are causally associated with the intervention, the intervention will be discontinued. There are no defined discontinuation criteria for parents. They have the option to terminate participation in the trial as well as EPP support at any time.

#### 2.5.3. Strategies to Improve Adherence to Interventions

Questionnaires will be used to check whether the EPP employees work according to the mentalization-based team approach. The ability to mentalize one’s own role and their role in the team will be assessed 3 times during the duration of the study with the help of the AMBIT Integrative Measure. Additionally, all contacts between the EPP team members and the families will be documented in a visit log book filled out by the EPP employees. All team supervisory sessions are recorded in a supervision log book by the supervisors, documenting all cases discussed. In addition, the EPP employees will evaluate their work when they have closed a case in terms of certain criteria with the help of the “Psychotherapists‘ work involvement scales” (PWIS [35]). The questions capture aspects such as how good the relationship with the family was, how well the employee was able to empathize with the situation of the family, and whether the supervision was helpful. The adherence of the supervisors to the MBT approach will additionally be controlled by the MBT supervision of the supervisors (Meta-Supervision).

#### 2.5.4. Relevant Concomitant Care Permitted or Prohibited during the Trial

Standard EPP support will include regular home visits, offers of voluntary group meetings (such as meetings at cafés with other young mothers), accompanied medical visits, or visits to authorities. All active and passive elements in the EPP are allowed to be used during the “EPP-plus” approach, e.g., team meetings, video-based interventions, regular additional non-case supervision, and cooperation with psychotherapists and psychiatrists.

### 2.6. Outcomes

The primary outcome variable is the change in the parent-reported Parental Stress Inventory (PSI, [36]) scores from pre- to postintervention. A high parental burden can be considered a risk factor for dysfunctional parenting behavior. The PSI is a well-established 48-item self-report questionnaire for caretakers and can be utilized as a screening tool for dysfunctional parent–child interactions [37]. Good psychometric properties have been reported for the EBI, with reliability scores ranging from α = 0.95 for the total score to α = 0.93/0.91 for the domain scores. The validity of the instrument has been examined in several studies that have reported substantial associations between EBI scores and various indicators of parental stress [37].

As secondary outcomes, more detailed assessments of the parent and children’s health, as well as parent–child interactions, will be conducted when the child is aged 12, 18, 24, or 30 months. Secondary parental outcomes are the total and subscale scores of the Adult & Adolescent Parenting Inventory (AAPI-2, [38]), parental health (Patient Health Questionnaire, PHQ, [39]), child abuse potential (Child Abuse Potential, CAPI, [40]) and the level of social support (Social Support Questionnaire, Fsozu, [41]). The internal consistency of the depression scale of the German PHQ is given as α = 0.88 [42]. The internal consistencies of the main scales and the total score of the FSozu are between α = 0.81 and α = 0.93 [41]. The reflective functioning of the parents will also be recorded before (T0) and after (T1) the intervention as well as during the follow-up (T2) using the Parental Reflective Functioning Questionnaire (PRFQ, [43]). Estimates of internal consistency were 0.70, 0.82, and 0.75 for the three subscales [43].

The categorical and psychometric diagnosis of expected moderators of therapy success will be carried out at T0 by two clinical interviews: the International Personality Disorder Examination (IPDE, [44]) and the International Neuropsychiatric Interview (MINI DIPS, [45]). The interrater reliability for IPDE predominantly lies above r = 0.70 [44]. Regarding the MINI DIPS, studies on the quality criteria indicate good interrater reliability for the major diagnostic categories of anxiety disorders, mood disorders, somatoform disorders, eating disorders, sleeping disorders, alcohol and substance disorders, and for all specific psychiatric disorders, and the exclusion of psychiatric disorders [46]. As a further moderator variable, the early life maltreatment experiences of the caregiver will be assessed with the help of the Childhood Experiences of Care Abuse Questionnaire (CECA-Q, [47]) and additionally with the Childhood Trauma Questionnaire (CTQ, [48]) and Maltreatment and Abuse Chronology of Exposure Interviews (MACE, [49]) in a subsample. The CECA.Q shows satisfactory reliability and validity as a self-report measure for adverse childhood experiences. Satisfactory internal scale consistency was achieved on the CECA.Q for antipathy (α = 0.81) and neglect (α = 0.80) scales [47]. The internal consistency of all CTQ scales (apart from physical neglect) was high (Cronbach’s α ≥ 0.89) [48]. The criteria for convergent validity in the MACE were correlation coefficients in the range of 0. 6–0.8; reasonable evidence of satisfying convergent validity was found [49].

All three measurements have shown satisfactory internal scale consistency.

The subsample will be determined at an advanced stage of the study. From this point on, all newly screened and included families will be informed about the additional data collection and can agree or decline it. The duration of contact between a team member and the parents will also be recorded to take this parameter into account in the statistical analysis.

Secondary outcomes of the children will include the parent-reported scores on the Infant-Toddler Social and Emotional Assessment (BITSEA, [50]) and the child’s temperament, assessed with the Infant Behavior Questionnaire (IBQ, [51]), as well as objective evidence of child maltreatment (injuries, hospital stays, and out-of-home accommodations) reported by the EPP staff. The latter will be logged by the EPP employee as part of their visit logs. Specific questions will be asked about hospitalizations or accidents since the last visit. These incidents are not counted as serious adverse events (sEAS). The BITSEA demonstrated acceptable internal consistency, test-retest reliability, and validity relative to other parent-report checklists [52]. Regarding the IBQ reliability, it was at acceptable levels for all subscales, ranging from 0.70 to 0.90 [53].

Additionally, a video-based diagnostic instrument will be used at T0 and T2 to measure the quality of parent–child interactions. In the context of attachment theory and research, due to the central importance of parental sensitivity, various methods have been developed to determine the quality of the relationship of the mother–child dyad. The Infant CARE Index is a video-based instrument for assessing the quality of infant–adult relationships [54]. To date, it is the only analytical method for early childhood (0 to 15 months) to identify covertly hostile behavior in adults and distinguish between the real and simulated cooperation of infants. Simulated cooperation thereby means that the child feigns cooperation because it has learned that certain behavior is desired by the interaction partner (e.g., fake smile). Its validity and reliability have been proven in numerous international studies. It is based on the systematic evaluation of a 3 min videographed game interaction. The Care Index provides a global score which allows a risk evaluation for child abuse [54].

The secondary endpoints for the EPP team members are the change in “reflective functioning” (reflection on their own role and their role in the team, adapted according to the “AMBIT Integrative Measure”, [55]) and stress experiences (Trier Inventory for the Assessment of Chronic Stress, TICS, [56]). The internal consistencies of the TICS scales range between 0.84 and 0.91 (M = 0.87), and the Rasch reliabilities between 0.78 and 0.89 (M = 0.83). The procedure has good profile reliability (0.72) [56].

### 2.7. Participant Timeline

**Figure 1:**
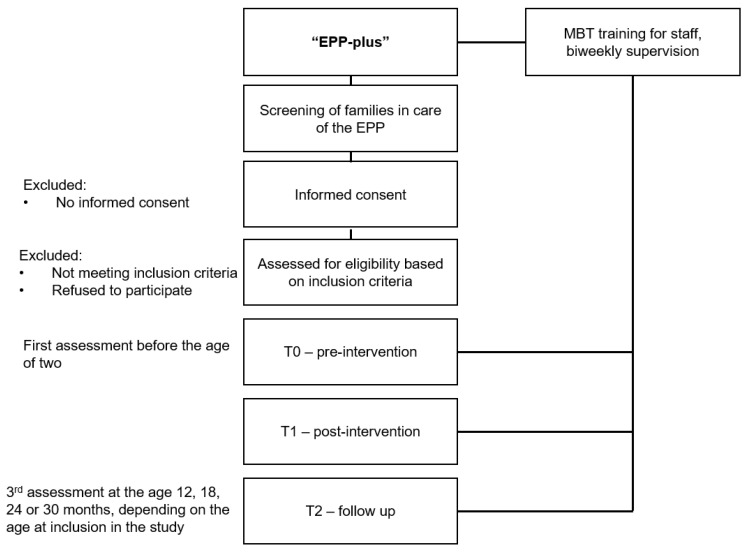
Study Flow Chart.

The flow of participants from recruitment through the end of the study is shown in Figure 1. Children and primary caregivers will be systematically assessed at T0 (preintervention), shortly after the parent has agreed to participate in the study, and at the end of the intervention carried out by the EPP staff (T1, postintervention). The timing of T1, however, will be different for each subject since the duration of the intervention is not standardized but depends on the individual needs and wishes of the family. A follow-up assessment will take place when the child is 12, 18, 24, or 30 months old, depending on the age at first assessment. If the child is ≤4 months old when the EPP team first assists, the assessment will be carried out when the child is 12 months old (T2); if the child is already older than 4 months old, the assessment will be carried out when the child is 18 months old (T2); if the child is already older than 12 months, the assessment will be carried out when the child is 24 months old (T2); and if the child is already older than 18 months, the assessment will be carried out when the child is 30 months old (T2). In the preintervention period, an EPP employee will have visited families a maximum of 5 times (corresponding to T0). The end of the intervention is defined by the end of support by the EPP (corresponding to T1). Moderators of treatment outcomes and sociodemographic data will be assessed at T0. Participants will be paid EUR 50 for each assessment session.

### 2.8. Sample Size

In the current study, we will analyze whether there is a statistically significant change in the primary outcome (PSI score for each parent) from T0 (preintervention) to T2 (follow-up assessment, paired comparison). With a sample size of 67 parents (with data at both time points), a power of 80% can be achieved (two-sided significance level of 0.05; one-sample t-test) for an effect of at least d = 0.35 (standardized difference in the mean; calculated with G * Power 3.1.9.6). Effects of this magnitude were observed for an MBT team approach in mothers (Suchman et al., 2013, moderate effects on caregiving behavior and child behaviors (d > 0.2), [57]) and are therefore also expected for the new “EPP-plus” approach. Taking into account a loss to follow-up rate of up to 20% (missing values in the primary outcome measure at FU), at least 84 study participants should be recruited.

### 2.9. Recruitment

Patient recruitment will take place in Aachen, Frechen (a small town near Aachen), and the surrounding areas. We expect that approximately 50% of all parents will be willing to participate in the study and will consent to the study assessments; thus, recruitment at the two sites will guarantee that the required sample size will be achieved within 24 months of recruitment. The drop-out rate until 1-year follow-up is estimated to be 20%, according to Suchman et al. (2013) [57]. Before the start of the study, the EPP in Aachen cared for an average of 249 parents per year (2016: 255; 2017: 231; S018: 260). However, due to the COVID-19 pandemic, recruiting difficulties have occurred, so recruitment proceeded much slower in some phases, to the point that a study design change became necessary (see also [8]). Participants are paid EUR 50 for each assessment session.

### 2.10. Plans for Assessment and Collection of Outcome Data

There are three measurement time points. All assessments will take place during home visits and be performed by the study staff (psychologists). All psychometric questionnaires will be completed on mobile devices, and the answers will be recorded with LimeSurvey software (Version 3.28.58). Clinical interviews will be carried out by a study employee who is trained in the procedures. In addition to the outcomes, sociodemographic data (such as partnership status and housing situation) and the socioeconomic status of the family will be collected according to the GEDA study by the Robert Koch Institute [58] and via a case report form (CRF). An overview of the diagnostic tools and when data will be collected can be found in Table 2.

### 2.11. Ethical Approval

The trial was approved by the local Research Ethics Committee at the University Hospital RWTH Aachen (reference EK 221/19) and will be carried out in accordance with the Declaration of Helsinki. The study was registered at the German Clinical Trials Register (DRKS00022075 on the 8 July 2020). Written, informed consent to participate will be obtained from all participants, both parents and EPP staff.

### 2.12. Statistical Methods for Primary and Secondary Outcomes

The effects of the “EPP-plus” are assessed in a pre-post comparison using the PSI scores with a one-sample t-test with a two-sided significance level of 5%. The primary analysis is based on the intention-to-treat (ITT) principle, which aims to include all subjects enrolled in the feasibility study. The analysis of the secondary results is carried out analogously. Exploratory analyses will investigate which family-level risk factors are associated with the magnitude of change in PSI, using linear models and considering the presence of socioeconomic disadvantage (e.g., unemployment, overall socioeconomic status), mental risk characteristics (e.g., mental/physical health, domestic violence), teenage motherhood, immigrant status, parental ELM (assessed by CECA-Q), AAPI-2 pre-intervention total score, age and gender (of parent and child), and child responsiveness (Infant Care Index).

The feasibility of the “EPP-plus” approach will be analyzed descriptively, using frequencies and proportions for nominal or ordinal variables or medians and interquartile ranges for quantitative variables. For feasibility, we will consider the median number of visits per family, the number of supervision sessions, and attendance by the EPP staff to supervision sessions. In addition, acceptance of the “EPP-plus” approach by the staff members and their work-related stress levels will be analyzed at the start, midterm, and end of the trial by linear mixed models accounting for the clustering of team members. Furthermore, potential staff- and team-related factors contributing to the outcome of the “EPP-plus” approach (such as job experience, professional background, etc.) will be analyzed.

All safety data, including adverse events, are analyzed descriptively in the Safety Population (SP), which includes all families who were visited at least once by a team member of the “EPP-plus” team.

### 2.13. Dissemination Plans

The results will be published in the form of articles in peer-reviewed scientific journals. There are no publication restrictions resulting from funding. After all results are published, the anonymized data will be available on demand. Moreover, a website will be developed to provide information about the project and the current status of the results to the interested public.

## 3. Discussion

This study protocol presents a non-randomized, open-label, single-arm trial investigating the feasibility of implementing a mentalization-based integrative team approach (MB-TA) aimed at reducing the risk of maltreatment among hard-to-reach parents in an already existing welfare system. On the basis of convincing evidence that mentalization-based approaches help professionals feel empowered and more confident in working with hard-to-reach patients or clients [15], the present approach focuses on strengthening the competencies of the primary care system so that the risk of maltreatment in burdened families can be reduced. The sample to be surveyed should meet the risk factors for child endangerment since the typical clientele of the EPP shows a high burden (low SES, mental disorder diagnosis, extreme parental stresses, and overload). Additionally, before proposing this study, sixty-nine percent of the EPP clients stay in treatment for less than three months, indicating major problems for the continuity of support [33]. This study’s inclusion criteria will additionally ensure that participating parents have at least one risk factor associated with an increased risk for child maltreatment [2,6,10,11,29]. However, the exact potential for child endangerment in the sample cannot be determined at this time. Self-report questionnaires will be used to measure whether parental risk factors can be reduced through EPP assistance. Self-report measures can be biased but allow data to be collected from a large number of individuals. Moreover, there are hardly any objective measures for the variables collected in this study. For example, parents’ own experiences of abuse can only be requested retrospectively. Less parental stress is significantly associated with fewer inadequate parenting behaviors, as well as child maltreatment [6], which is why the PSI score was chosen as the primary outcome. Actual maltreatment reported by the EPP is used as a secondary outcome since no objective data on this were available before the intervention, and therefore, no pre-post effects can be examined. To our knowledge, the study represents the only feasibility study investigating the effects of mentalization-based psychiatric/psychotherapeutic supervision on childrearing practices and the risk of maltreatment to date. Consequently, the present study gives profound consideration to the repeatedly formulated necessity to develop effective intervention approaches to reduce child maltreatment by empowering the care system. There is a specific need to implement and evaluate intervention approaches within existing welfare systems that meet specific criteria for quality of service, including multi-professional teams, cooperation with community health and welfare services, and work via home visits since these criteria have already been identified as necessary for treatment success [1,8,28]. However, early support by itself seems ineffective at reducing the risk of child maltreatment [24]. It seems of major relevance for staff workers to pay special attention to the known parental risk factors to reduce child maltreatment. At this point, however, EPP staff reports a lack of knowledge. Thus, a team-based approach providing the staff with special knowledge and skills is urgently needed to strengthen the competencies of EPP staff and, thereby, the chance to provide continuous care for burdened families [59]. Mentalization-based intervention programs have emerged in working with hard-to-reach clients since adequate mentalizing allows us to interact in a highly adaptive manner and to take the needs of others into account. One study showed that it allows healthcare professionals to acquire empathy and understanding for their clients and increases consistency in their teamwork [26]. Therefore, the MB-TA seems very promising to support EPP staff and enhance networking between agencies. If the current metalization-based intervention program is feasible, then the effectiveness of the approach should be verified with the help of randomized controlled studies. In case of a confirmatory result, the findings will have important implications for community service and clinical work. The MB-TA can be widely integrated into existing welfare systems. This offers the advantage of a comparatively easy and time-saving implementation process. There is no need to set up a new team of helpers, and existing team structures do not have to be restructured and can simply be supplemented with MB-TA team training and subsequent regular mentalization-based supervision. However, we are currently unable to estimate the exact costs of the intervention. However, the data on cost-benefit analyses show that the consequences of child maltreatment are considerable. A comparison shows that the prevention costs are comparatively minimal compared to the consequential costs of child endangerment. To be more precise, in the area of early intervention, the costs of unprevented child abuse are 60 (moderately calculated) to 159 (pessimistically calculated) times higher than the costs of EPPs. So even if the MB-TA intervention causes higher costs than treatment as usual, it should be cost-effective if there is evidence of its higher effectiveness [60]. In addition to the cost-benefit analysis of German EPPs, Finnish researchers have examined the economic aspects of team supervision and were able to show that team supervision was efficient in this respect [61].

## 4. Conclusions

Children of parents with severe psychosocial risk factors, such as low education levels, low family income levels, social isolation, high levels of parenting stress, mental illness, and a history of early abuse, are prone to maltreatment and neglect. Although home visiting programs have generally shown small but significant overall effects on the prevention of child maltreatment, at-risk families with severe strain do not seem to benefit sufficiently from this support. A crucial factor for success seems to be the quality of the service system, especially supervision and training, which emerged as important implementation factors. In particular, the inclusion of a reflective supervision component resulted in greater program effectiveness. The presented study, therefore, aims to contribute to the further improvement of EPPs by adding mentalization-based team supervision to the already existing welfare service of the German EPP. We hypothesize that mentalization-based supervision supports the staff in developing a more empathetic and consistent approach to challenging clients and a better understanding of parental mental states to keep the families in the care program, thus reducing the risk of childhood abuse, maltreatment, and neglect. Initial experiences with mentalization-based supervision have already shown a high level of satisfaction among EPP staff. In the evaluations that have been carried out to date, employees have also stated that they benefit from the supervision service. Finally, if feasibility emerges from this study, the MB-TA approach can then be incorporated into future intensive evaluation and, with corresponding proof of effectiveness, sustainably improve the EPP in Germany.

## Figures and Tables

**Table 1:** Inclusion and exclusion criteria.

Inclusion Criteria	
Parent/caregiver	Must be supported by the EPP Score > 4 on at least one of the five subscales of the Adult Adolescent Parenting Inventory (AAPI version 2.1) Or the presence of socioeconomic disadvantage Or the presence of parental risk characteristics Or teenage motherhood Written informed consent provided by the caregiver
Child	<24 months of age
Exclusion criteria	None

**Table 2:** Plans for the assessment and collection of outcome data.

Diagnostic Tools	Subject	T0	T1	T2
Sociodemographic data	Parent	X		X
MINI DIPS	Parent	X		
IPDE	Parent	X		
CECA-Q	Parent	X		
AAPI-2 (Version A)	Parent	X		X
AAPI-2 (Version B)	Parent		X	
Infant Care Index	Parent	X		X
PHQ	Parent	X	X	X
PSI	Parent	X	X	X
CAPI	Parent	X	X	X
PRFQ	Parent	X	X	X
F-Sozu	Parent	X		X
BITSEA	Parent			X
IBQ				X
PWIS	EPP staff		X	
Visit log	EPP staff		X	
AMBIT	EPP staff	3 times during the course of the study		
TICS	EPP staff	3 times during the course of the study		
Subgroup:				
Saliva (Epigenetic)	Participant	X		
CTQ	Participant	X		
KERF (MACE)	Participant	X		

## Data Availability

Not applicable.
